# Influence of nano-VC on the structural and magnetic properties of MnAlC-alloy

**DOI:** 10.1038/s41598-021-93395-2

**Published:** 2021-07-14

**Authors:** Vitalii Shtender, Henry Stopfel, Daniel Hedlund, Dennis Karlsson, Rajasekhar Pothala, Björn Skårman, Fredrik Olsson, Hilmar Vidarsson, Gabriella Andersson, Peter Svedlindh, Martin Sahlberg

**Affiliations:** 1 Ångström Laboratory, Department of Chemistry, Uppsala University, Box 538, 751 21 Uppsala, Sweden; 2 Department of Materials Science and Engineering, Uppsala University, Box 35, 751 03 Uppsala, Sweden; 3 Department of Physics and Astronomy, Uppsala University, Box 516, 751 20 Uppsala, Sweden; 4 Höganäs AB, Bruksgatan 35, 263 83 Höganäs, Sweden

**Keywords:** Materials science, Chemistry, Inorganic chemistry, Materials chemistry

## Abstract

Alloys of Mn_55_Al_45_C_2_ with additions of VC nano-particles have been synthesized and their properties evaluated. The Mn_55_Al_45_C_2_(VC)_*x*_ (*x* = 0.25, 0.5 and 1) alloys have been prepared by induction melting resulting in a high content of the ferromagnetic τ-phase (> 94 wt.%). Powder X-ray diffraction indicates that nano-VC can be dissolved in the alloy matrix up to 1 at.%. On the other side, metallography investigations by scanning electron microscopy and scanning transmission electron microscope show inclusions of the nanosized additives in the microstructure. The effect of nano-VC on the grain and twin boundaries has been studied by electron backscattering diffraction. The magnetization has been measured by magnetometry up to 9 T while the domain structure has been studied using both magnetic force microscopy as well as Kerr-microscopy. For nano-VC contents above 0.25 at.%, a clear increase of the coercive force is observed, from 57 to 71 kA/m. The optimum appears to be for 0.5 at.% nano-VC which shows a 25% increase in coercive force without losing any saturation magnetization. This independent increase in coercivity is believed to originate from the nano-VC reducing the overall magnetic domain size. Overall, we observe that addition of nano-VC could be an interesting route to increase the coercive force of MnAl, without sacrificing saturation magnetization.

## Introduction

The mechanical-to-electrical energy conversion or vice versa is a key technology for the renewable energy and electro-mobility markets. The generators and converters used for example in electric cars or wind turbines require the use of permanent magnets. The permanent magnet market nowadays is roughly comprised of hexagonal ferrites and the rare-earth neodymium-based magnets, where Alnico and SmCo_5_ based magnets only play a minor role. After the rare-earth crisis in 2011^1^, increasing attention has been placed on finding the so-called gap permanent magnets^2^ with performance exceeding those of hexagonal ferrites. One potential candidate is MnAl, which has enough anisotropy^3^ to be a permanent magnet of gap performance, i.e. to be able to supersede hexagonal ferrites but not rare-earth magnets. In the Mn–Al phase diagram^4^, there is only a single ferromagnetic phase, τ-MnAl, which is metastable^5^. The compound was discovered by Kono in 1958^6^ and found to be ferromagnetic by Koch^7^ in 1960 but has still not been realized as a commercial product despite promising properties such as high saturation magnetization (${M}_{S}$), high Curie temperature (${T}_{C}$), high magnetocrystalline anisotropy (${K}_{1}$) and a good maximum theoretical energy product (${(BH)}_{max}$).

There are mainly two reasons why MnAl has not yet been commercialized. One is that before the rare-earth crisis, there was little to no incentive to develop gap magnets. The other is a more fundamental material issue on the microstructural level. The celebrated Brown’s paradox^8^ on the coercive force (${H}_{c}$) of a material gives insight on why the coercive force never reaches even nearly the upper limit of the anisotropy field. According to Kronmüller’s equation^9^,

$${H}_{c}=\frac{2\alpha {K}_{1}}{{\mu }_{0}{M}_{S}}-{D}_{\mathrm{e}\mathrm{f}\mathrm{f}}{M}_{S,}$$

this is mainly due to the microstructure’s ability to pin and nucleate the field. In Kronmüller’s equation $\alpha$ is a dimensionless factor (for most materials <  < 1), ${\mu }_{0}$ is the vacuum permeability and ${D}_{\mathrm{e}\mathrm{f}\mathrm{f}}$ a local anisotropy constant related to the shape. In a well-controlled microstructure, $\alpha$ takes the value of 0.1–0.3. In τ-MnAl, much struggle has been placed on achieving phase pure samples through a viable synthesis method. This can be done through, for instance, drop synthesis^10^, strip casting^11^, gas atomization^12^ and melt spinning^13^. It is possible to stabilize the metastable τ-phase and thus improve purity through extra alloying with for instance C^14^ or Ga^15^. For a general overview of synthesis methods see e.g. Chaturvedi et al*.*^16^. Two recent studies about phases in the Mn–Al–C systems were presented by Bajenova et al*.*^17,18^.

In a recent study, nanoparticles of rare earths (*RE*) were added in MnAl (but without carbon) as (Mn_0.5+*x*_Al_0.5-*x*_)_100-*y*_*RE*_*y*_ (0.01 ≤ *x* ≤ 0.09, 0.1 ≤ *y* ≤ 1.0)^19,20^, which created nanoprecipitates of ~ 20 nm size that helped to pin domain walls and thus realizing a coercivity as large as 420 kA/m (5.43 kOe). We therefore, chose Mn_55_Al_45_C_2_ as base material for our investigations since it is more stable and the performance is more promising compared to the C-free τ-MnAl. The goal was to prepare Mn_55_Al_45_C_2_ with well dispersed nanoparticles to create pinning sites for domain walls. To achieve this, we added different amount of nano-VC to the Mn_55_Al_45_C_2_ base compound to elucidate its effect on the microstructure and magnetic properties.

## Experimental details

Mn_55_Al_45_C_2_(VC)_*x*_ (*x* = 0, 0.25, 0.5, 1 and 3.5) alloys (~ 15 g each) were synthesized by induction melting. High purity raw materials have been used as starting materials for synthesis of the Mn_55_Al_45_C_2_(VC)_*x*_ alloys: Mn powder (Mn powder from Höganäs AB, purity 99.9%), Al (Gränges SM, purity 99.999%), C (Highways international, purity 99.999%), VC nanopowder (High purity VC nanopowder from INSCX exchange, purity > 99%, size 80–100 nm). Firstly, powders of Mn and nano-VC were weighed, well mixed and pressed into pellets in a glovebox. Appropriate amounts of Al and C were put together with the Mn_55_(VC)_*x*_-pellets in an Al_2_O_3_-crucible. Afterwards the crucible was transferred to a high frequency induction furnace and vacuum pumped for 1 h. The melting was done by introducing 5 N Ar gas to have an atmosphere of 400 mbar. At the highest temperature (1300–1500 °C) the temperature was kept for 1–3 min to form a homogeneous solution. Finally, the furnace was switched off, followed by natural cooling to room temperature. The total mass loss during synthesis was less than 2 wt.%. In the event of larger losses, more Mn was added to compensate as for these alloys only Mn evaporates during melting.

All samples were characterized using a Bruker D8 powder X-ray diffractometer with a Lynx-eye position sensitive detector and Cu-*K*α radiation on a zero-background single crystal Si sample holder. Phase analysis and Rietveld refinements of the X-ray data were done with the GSAS suite software^21^. For the Rietveld refinement of the PXRD we have used following information from literature^14^: the main τ-phase is a tetragonal L1_0_ superstructure (*P*4/*mmm*, AuCu-Ι type) with interstitial of C at [1/2 1/2 0], γ_2_ phase (*R*3*m*, Al_8_Cr_5_-type) and VC (*Fm﻿﻿-3m*, NaCl-type).

Microstructural evaluations were carried out using scanning electron microscopy (SEM), energy-dispersive X-ray spectrometry (EDS), electron backscattering diffraction (EBSD), magnetic force microscopy (MFM) as well as magneto-optical Kerr effect (MOKE) microscopy. Samples were cut in an Accutom-50 and subsequently mounted in Levofast resin and bakelite PhenoCure™, grinded and polished down to 1 µm by a standard process. The polishing was done with OPS-S non-drying colloidal silica suspension for 5 min in order to improve planeness and for relief polishing. Before etching, the samples were analyzed with a light optical microscope (LOM) in a Zeiss Axio Imager M2m microscope. They were afterwards etched in a diluted modified Keller’s reagent: with HCl 6 ml, HNO_3_ 3 ml, HF 1 ml and H_2_O 290 ml. Samples were analyzed in a field emission scanning electron microscope (FE-SEM) Hitachi SU6600 equipped with an EDS system (Bruker EDX XFLASH 5010). Furthermore, the EBSD experiment was done using a Zeiss Merlin equipped with a Nordlys Max detector and evaluated by the AZtec HKL software. Characterization at high magnification was done using an FEI Titan Themis 200 scanning transmission electron microscope (STEM) equipped with SuperX EDS and spherical aberration corrector. The TEM specimen was prepared by the in-situ lift-out technique in a Zeiss Crossbeam 550 focused ion beam scanning electron microscope (FIB-SEM). The surface was protected with Pt and a final polishing step using a 5 kV ion beam was utilized to minimize beam damage from the preparation.

Magnetic force microscopy (MFM) was performed using a Bruker Dimension Icon system. Images were processed by the software GWYDDION^22^ using background correction and flattening. Magnetic hysteresis curves were measured at 300 K using a Quantum Design PPMS with a maximum applied field of 9 T. The samples were corrected for demagnetizing effects.

An evico magnetics Kerr microscope, with motion correction since the forces on the samples are rather strong, was used for magnetic domains observation. Room temperature Kerr microscopy studies were carried out in longitudinal geometry, i.e. applying an in-plane magnetic field parallel to the plane of incidence. In this configuration, out-of-plane components are dominating the magnetic contrast^23^.

## Results and discussion

### Synthesis and phase analysis

**Figure 1:**
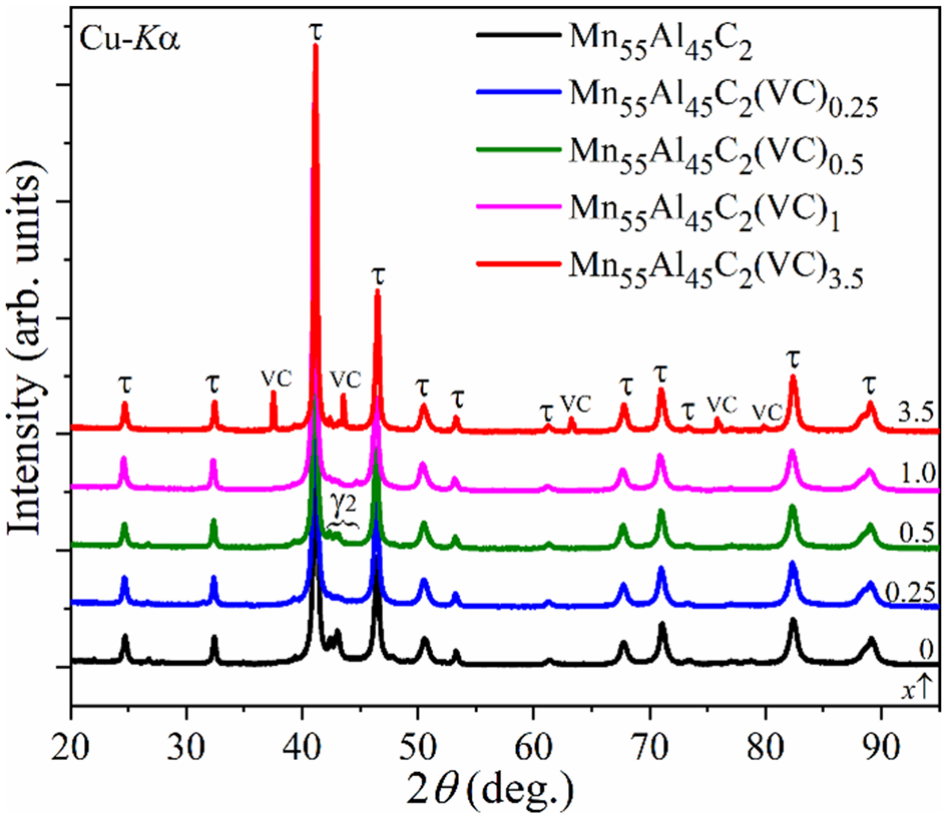
Comparison of the PXRD patterns for the as synthesized Mn_55_Al_45_C_2_(VC)_*x*_ (*x* = 0, 0.25, 0.5, 1 and 3.5) alloys.

Previous investigations on the carbon stabilized τ-Mn_55_Al_45_ phase (Mn_55_Al_45_C_2_ made by drop synthesis)^10,24,25^ showed great purity and promising magnetic properties. However, despite being able to obtain close to 100% phase pure τ-MnAl the material still has challenges, mainly in terms of its microstructural properties which need to be improved to compete with ferrites. In this investigation, nano-VC additive has been selected in an attempt to achieve this. It should be taken into account that all samples in this work were synthesized by direct induction melting and all further investigations were done on the as-prepared samples. The Mn_55_Al_45_C_2_ alloy has been made as a reference for comparison. Investigation of the quality of Mn_55_Al_45_C_2_(VC)_*x*_ alloys has been done by powder X-ray diffraction (PXRD). Figure 1 presents a comparison of PXRD patterns for the Mn_55_Al_45_C_2_, Mn_55_Al_45_C_2_(VC)_0.25_, Mn_55_Al_45_C_2_(VC)_0.5_, Mn_55_Al_45_C_2_(VC)_1_ and Mn_55_Al_45_C_2_(VC)_3.5_ samples. Notably, peaks of VC are not visible by PXRD up to a concentration of 1 at.% of VC. A complete phase analysis of the synthesized and processed samples is presented in Table 1.

**Table 1:** Phase analysis, magnetization at 9 T and coercivity values of the studied Mn_55_Al_45_C_2_(VC)_*x*_ (*x* = 0, 0.25, 0.5, 1 and 3.5) alloys. Abbreviations used in the table: τ-Mn_55_Al_45_C_2_ and γ_2_-Mn_5_Al_8_.

No	Sample	Phase analysis			Magnetometry data	
		τ-phase parameters		Phase abundance (wt.%)	τ-phase parameters	
		$V$(Å^3^)	$c/a$		$M$(kA/m)	${H}_{C}$(kA/m)
1	Mn_55_Al_45_C_2_	27.599(3)	1.3039	85.0 τ + 15.0 γ_2_	541	57
2	Mn_55_Al_45_C_2_(VC)_0.25_	27.617(3)	1.3055	97.5 τ + 2.5 γ_2_	565	57
3	Mn_55_Al_45_C_2_(VC)_0.5_	27.620(4)	1.3049	94.0 τ + 6.0 γ_2_	544	71
4	Mn_55_Al_45_C_2_(VC)_1_	27.630(1)	1.3040	96.3 τ + 3.7 γ_2_	499	69
5	Mn_55_Al_45_C_2_(VC)_3.5_	27.816(1)	1.3060	86 τ + 5 γ_2_ + 9 VC	-	-

From the Rietveld refinement it was found that Mn_55_Al_45_C_2_ has 85 wt.% of τ-phase purity while doped Mn_55_Al_45_C_2_(VC)_*x*_ (*x* ≤ 1) alloys have greater purity (see in Table 1). From PXRD, the relative content of the τ-phase in the Mn_55_Al_45_C_2_(VC)_*x*_ alloys is very close to the drop synthesized^10^. Using C is thus important for stabilization of the τ-phase even when introducing nano-dopants. One sample with larger content of nano-VC (3.5 at.%) has been prepared to verify the solubility limit. Additionally, one alloy without carbon (sample not presented here) but with nano-VC has been made. However, the purity of the C-free sample dropped drastically (65 wt.% of τ-phase). It should be noted that the chosen additive even promotes and amplifies in combination with carbon the stabilization/formation of the τ-phase alloys.

### Microstructural analysis

#### SEM/LOM/EDX/STEM

The Mn_55_Al_45_C_2_(VC)_1_ alloy was selected for detailed microstructural analysis. SEM observations on this etched sample (see Supplementary information Fig. S1a) show that there exist clusters (regions) of striations aligned along preferential directions. Before etching, images were collected by LOM. The polarized LOM image (Figure S1b) gives a good insight into the grain size, orientations and its distribution, which is difficult to observe by ordinary LOM/SEM even after etching. The matrix (τ-phase) that exists in all samples is very homogeneous without large agglomerates of VC.

EDX analysis cannot confirm the existence of nano-VC in the alloy, since the particles are too small in comparison with the resolution of the instrument. However, EDX mapping reveals a homogeneous distribution of V and a few Al-rich regions within the τ-phase (Figure S2). In addition, the average contents of Mn and Al measured by EDX is Mn_55_Al_45_ (Mn-rich region) and Mn_39_Al_61_(Al-rich region) are in agreement with the τ-Mn_0.55_Al_0.45_C_0.02_ and γ_2_-Mn_5_Al_8_ phases, respectively. This is in good agreement with the results from PXRD.

**Figure 2:**
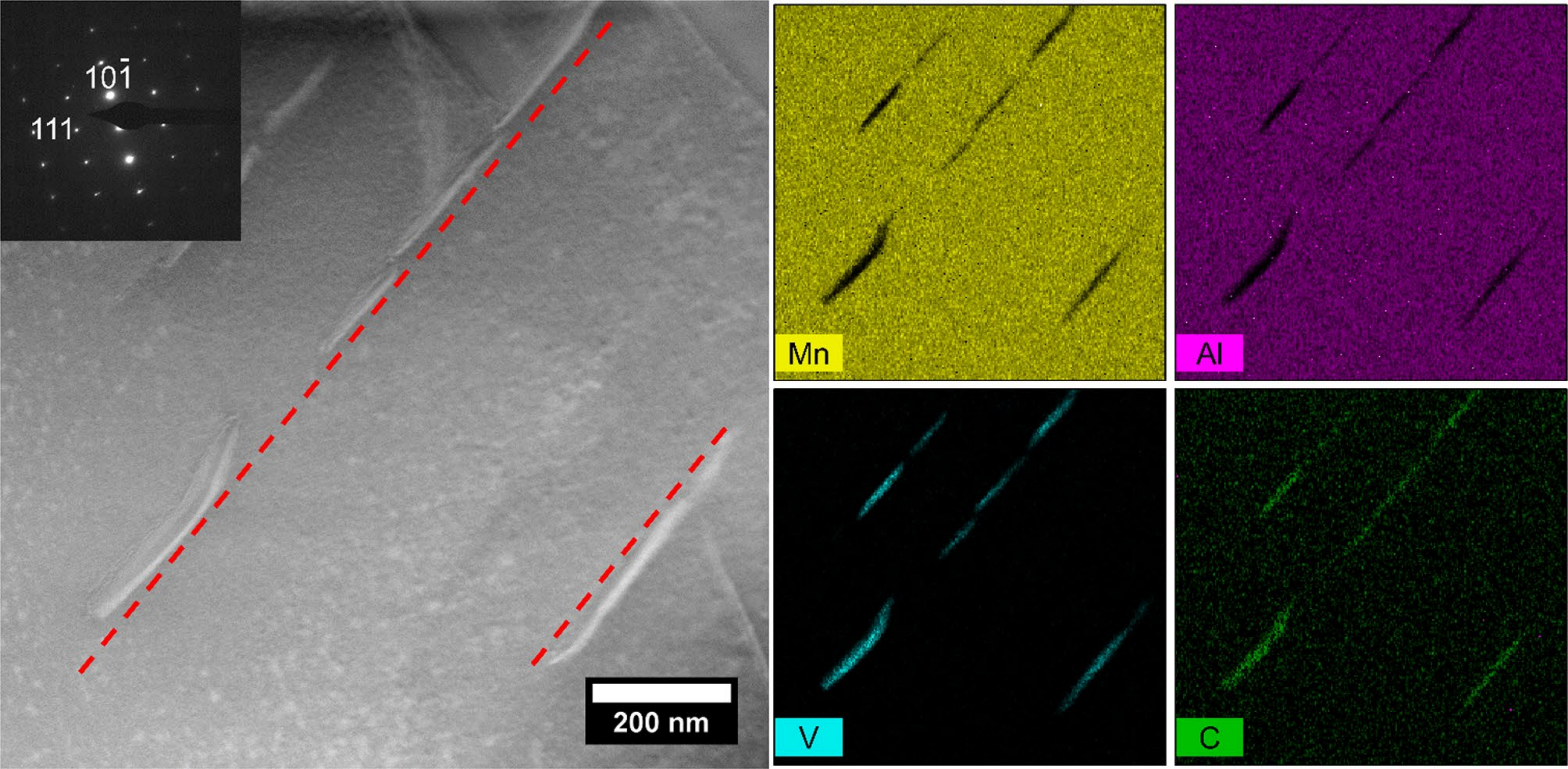
STEM image of the Mn_55_Al_45_C_2_(VC)_1_ sample, showing V-, C-rich precipitates forming in the τ-phase. The inset shows an electron diffraction pattern, indexed with the τ-phase oriented in the[1–21] zone axis. The red line indicates the [103] direction.

The presence of VC particles in the τ-phase was further investigated in the TEM. Figure 2 shows a magnified image of the τ-phase, where it can be seen that particles are present within the grain. The particles are elongated in one direction, forming needle-shaped precipitates with a length of around 200 nm and width of around 20–30 nm. These are rich in V and C, indicating the presence of nanocrystalline VC. As have been described earlier, these particles are found in preferential orientations according to the LOM and SEM images. The preferential orientation could also be seen in the TEM cross section, where the red line in Fig. 2 indicates the [103] direction. From these results, it is suggested that the particles have a preferred orientation in regard to the τ-phase, but the same orientation relationship was seen across twin boundaries in the TEM (Figure S3). Due to the high melting point of VC, it is expected that the added nanoparticles are distributed in the liquid phase, but remain as solid particles due to the high melting point and acting as nucleation sites for the τ-phase. Further investigation of this phenomenon is required to understand the solidification behavior of the alloy with VC, but is outside the scope of this study.

#### EBSD

**Figure 3:**
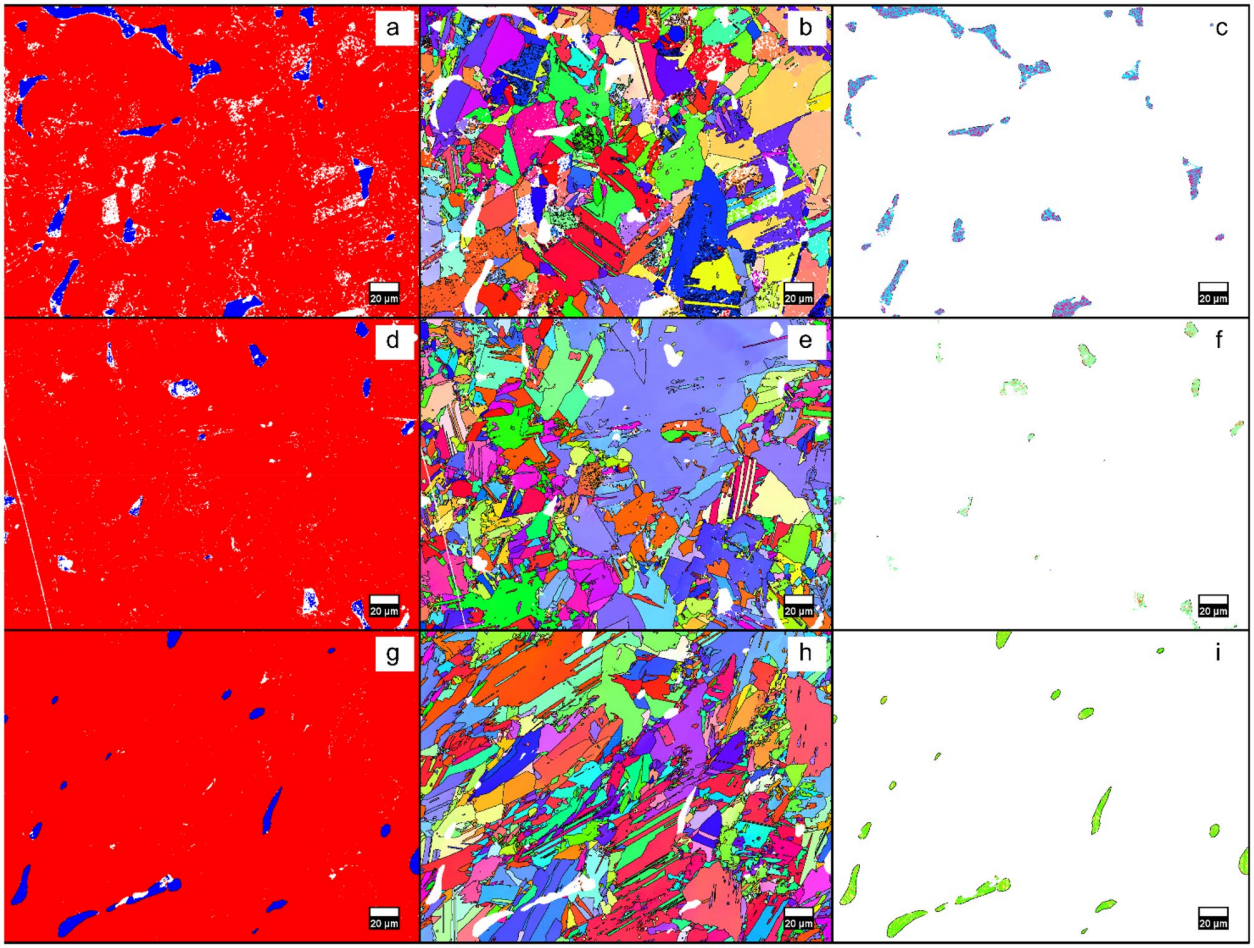
Phase maps with the τ-phase in red and the γ_2_-phase in blue obtained from EBSD measurements for the Mn_55_Al_45_C_2_ **(a)**, Mn_55_Al_45_C_2_(VC)_0.5_ **(d)** and Mn_55_Al_45_C(VC)_1_ **(g)** samples. Figures (**b,e,h**) show orientation maps for the τ-phase, and (**c,f,i**) show orientation maps for the γ_2_-phase.

Figure 3 shows the EBSD results for the Mn_55_Al_45_C_2_, Mn_55_Al_45_C_2_(VC)_0.5_ and Mn_55_Al_45_C(VC)_1_ samples. It can be seen in Fig. 3a,d,g that all samples contain a combination of the τ-phase (shown in red) with some γ_2_-phase (shown in blue) randomly distributed within the samples. Figure 3b,e,h,c,f,i show EBSD orientation maps of the τ-phase and the γ_2_-phase for the three samples. The τ-phase is randomly oriented, but all individual grains of the γ_2_-phase appear to be oriented in similar directions, which could originate from solidification if the γ_2_-phase solidifies with a preferred orientation in regard to the thermal gradient. In the last few years, much work has been done to elucidate certain features of the microstructure and its effect on the performance of τ-MnAl such as anti-phase defects^26^, dislocations^27,28^, interfaces^29^ and twins^26,27,30^. As many of these features are coexisting, often together with a difference in tetragonality (*c*/*a* ratio) of the τ phase, it is still not fully resolved which of these features have a negative impact on magnetic properties like magnetization, remanence and coercivity. Very recently, two studies^27,31^ were presented which show that for instance the coercivity can be negatively affected by twinning and that dislocations instead improve the coercivity.

**Figure 4:**
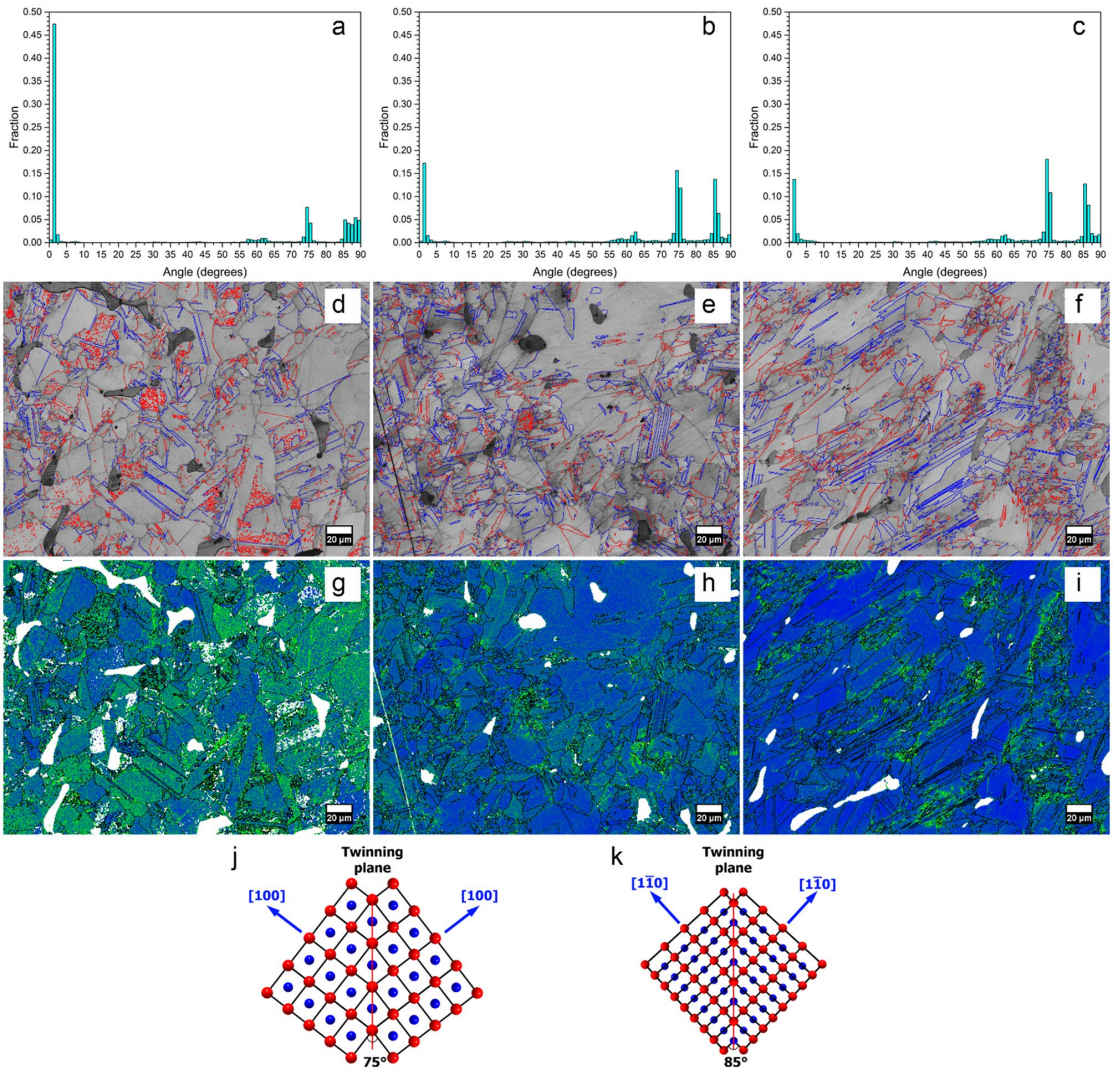
**(a–c)** show the grain boundary distribution for the Mn_55_Al_45_C_2_, Mn_55_Al_45_C_2_(VC)_0.5_ and Mn_55_Al_45_C(VC)_1_ samples and **(d–f)** show the corresponding <100> 75˚ and <110> 85˚ twins in red and blue. **(g–i)** show the local misorientation for the Mn_55_Al_45_C_2_, Mn_55_Al_45_C_2_(VC)_0.5_ and Mn_55_Al_45_C(VC)_1_ samples. **(j,k)** illustrate the <100> 75° and <110> 85° twin boundaries, respectively. For simplicity, an atomic ratio of Al:Mn = 1:1 was assumed; red atoms (corners) are Al while blue atoms (center) are Mn.

Figure 4 a-c shows the distribution of grain boundaries in the Mn_55_Al_45_C_2_, Mn_55_Al_45_C_2_(VC)_0.5_ and Mn_55_Al_45_C(VC)_1_ samples, respectively. It can be seen that a large amount of low angle grain boundaries (almost 50%) are formed in the sample without nano-VC, as well as a large fraction of grain boundaries with angles of 75˚ and 85˚ corresponding to <100> and <110> twins. With the addition of nano-VC, the amount of small angle grain boundaries decreases significantly to 17% and 14% for 0.5 at.% and 1 at.% respectively. Figure 4d–f show the distribution of the <100> 75˚ and <110> 85˚ twin boundaries in blue and red, respectively. Figure 4 g–i show the local misorientation for the Mn_55_Al_45_C_2_, Mn_55_Al_45_C_2_(VC)_0.5_ and Mn_55_Al_45_C(VC)_1_ samples, respectively. Furthermore, the coherent boundaries in the twins are illustrated in Fig. 4j,k for the <100> 75° and <110> 85° twins, respectively.

### Magnetic characterization

#### Magnetic measurements

**Figure 5:**
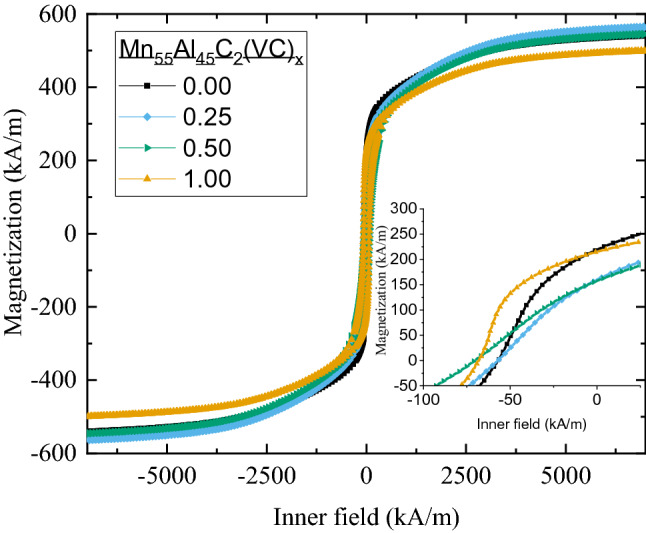
Magnetic hysteresis curves for Mn_55_Al_45_C_2_, Mn_55_Al_45_C_2_(VC)_0.25_, Mn_55_Al_45_C_2_(VC)_0.5_ and Mn_55_Al_45_C_2_(VC)_1.0_. Inset shows behavior near the coercive field.

Hysteresis measurements were performed up to 7200 kA/m (9 T) on samples of Mn_55_Al_45_C_2_, Mn_55_Al_45_C_2_(VC)_0.25_, Mn_55_Al_45_C_2_(VC)_0.5_ and Mn_55_Al_45_C_2_(VC)_1_ to investigate the magnetic properties. All curves were corrected for demagnetizing effects. The results can be seen in Fig. 5 and Table 1.

To start with, there is no strong correlation between the magnetization measured in an applied magnetic field of 9 T and the amount of τ-phase. Compare for instance Mn_55_Al_45_C_2_(VC)_0.5_ and Mn_55_Al_45_C_2_(VC)_1_, where first one has slightly less concentration of τ-phase compare to the last one (94.0 *vs*. 96.3 wt%, respectively). However, measured magnetization values show opposite trend (544 kA/m and 499 kA/m, cf. Table 1). Increasing the C content of (Mn_0.54_Al_0.46_)_100_C_*x*_ increases saturation magnetization^14^ slightly from carbon free to some carbon added, while too much decreases the saturation magnetization. This is however further complicated by the fact that the *c*/*a* ratio varies between the samples, which is also known to affect magnetization^32^. The τ-phase in all Mn_55_Al_45_C_2_(VC)_*x*_ (*x* = 0.25, 0.50 and 1.0) alloys has higher *c*/*a* ratio than in Mn_55_Al_45_C_2_, which also has been seen to affect both *H*_*A*_ and *T*_*C*_. This increase in *c/a* ratio is most probably due to differences in the C content of the samples.

The vanadium carbide seems to have more pronounced effect on the coercive force, which is varying between 57–71 kA/m. The optimum appears to be for 0.5 at.% nano-VC which shows a 25% increase in coercive force (from 57 to 71 kA/m) without losing any saturation magnetization.

#### MFM and Ker microscopy

**Figure 6:**
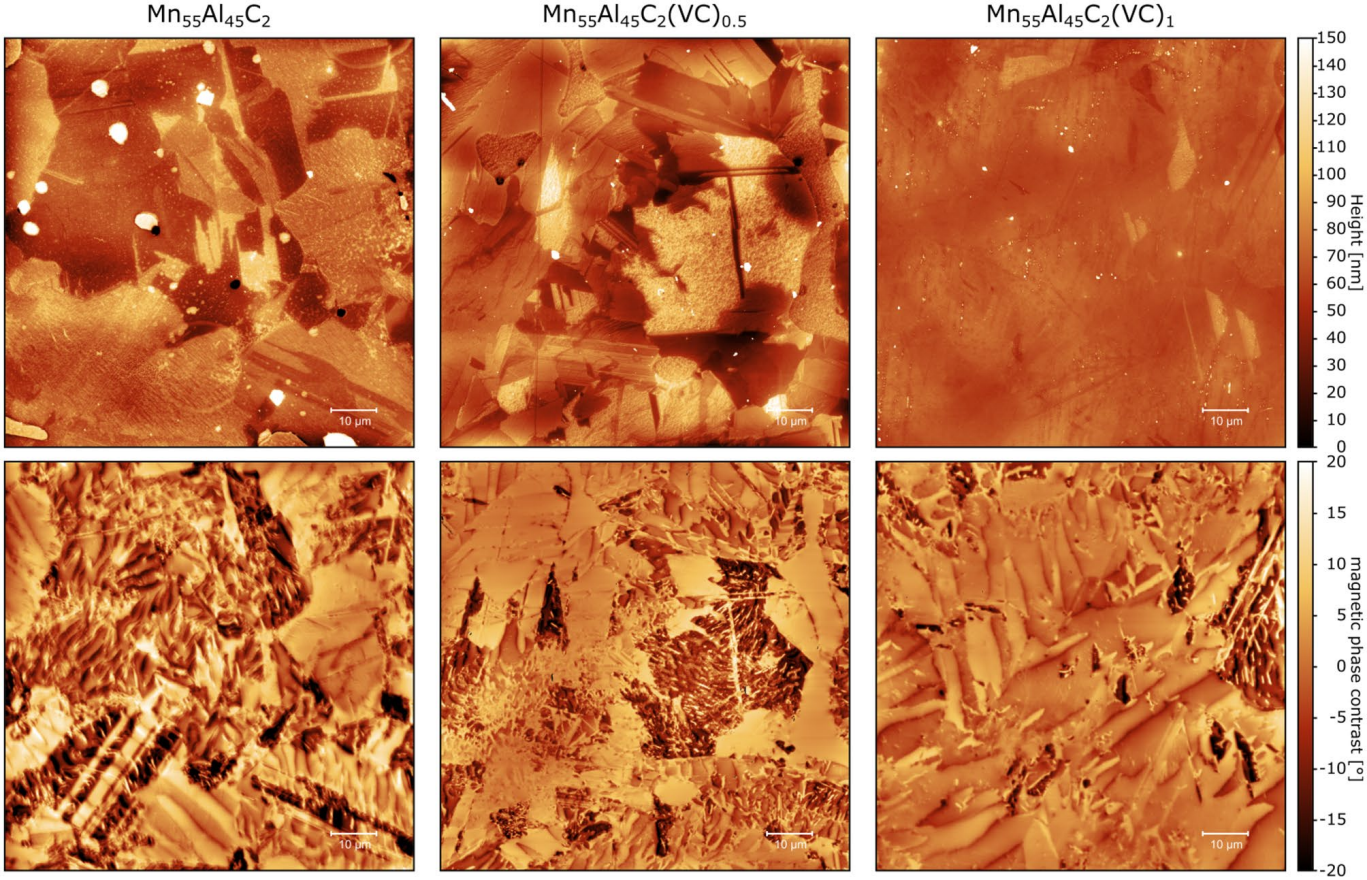
Representative results from MFM measurements of Mn_55_Al_45_C_2_, Mn_55_Al_45_C_2_(VC)_0.5_ and Mn_55_Al_45_C_2_(VC)_1_. First row shows the topography of the measured sample, while the second row represents the magnetic contrast illustrating the magnetic domain structure at the measured position.

We performed magnetic force microscopy (MFM) studies on the as-cast samples for Mn_55_Al_45_C_2_, Mn_55_Al_45_C_2_(VC)_0.5_ and Mn_55_Al_45_C_2_(VC)_1_ to investigate the influence of added precipitates on the magnetic microstructure. The as-cast samples were polished in the same way as it had been done for the SEM studies. The measurements were performed on a Dimension Icon system from Bruker. Each sample was investigated at different positions and one representative result is shown in Fig. 6.

Qualitatively, smaller domains can be seen for the samples with VC precipitates, to investigate the domain size quantitatively we use the stereological procedure^33^. For the stereological procedure, arbitrary line profiles are drawn through the domain image and the changes of the domain state along each line is recorded. The domain width is calculated as follows:

$$domain\,\,width=\frac{2\times\,\,line\,\,length}{\pi\,\,\times\,\,number\,\,of\,\,domain\,\,changes}$$

**Figure 7:**
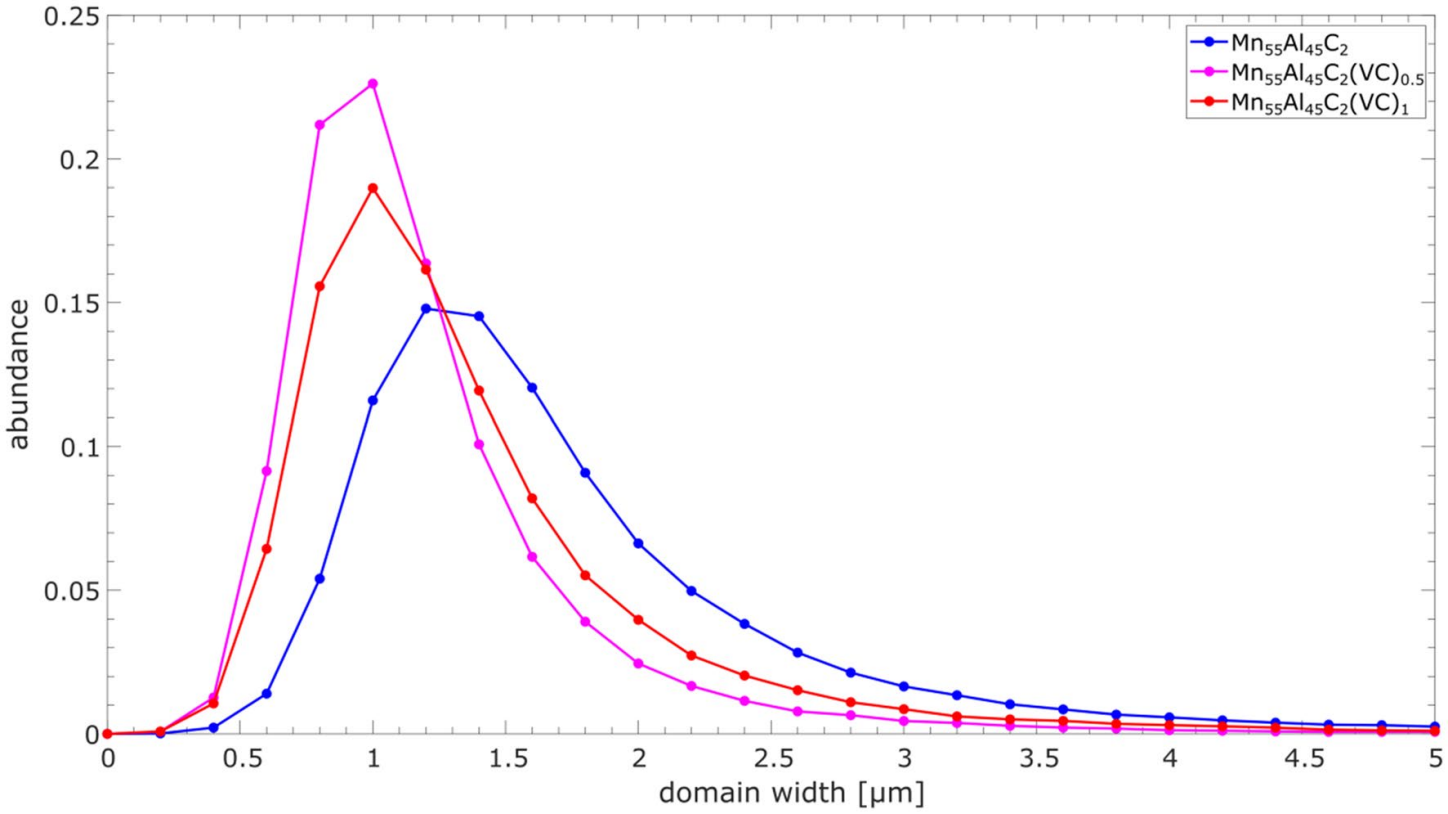
Domain width distributions for Mn_55_Al_45_C_2_, Mn_55_Al_45_C_2_(VC)_0.5_ and Mn_55_Al_45_C_2_(VC)_1_. A clear peak shift to lower domain width is visible in both the samples with 0.5% and 1% VC precipitates.

Applying this procedure to each domain image and averaging the results for one sample yield the domain width distributions presented in Fig. 7.

Comparing the distributions of Mn_55_Al_45_C_2_, Mn_55_Al_45_C_2_(VC)_0.5_ and Mn_55_Al_45_C_2_(VC)_1_ it can be seen that the samples with VC precipitates have a reduced domain width, verifying the more qualitative observations based on Fig. 6.

Investigating the Mn_55_Al_45_C_2_(VC)_1_ sample in more detail, we noticed at different locations a correlation between the domain appearance and precipitates visible in the topography. Figure 6 shows such a correlation between striations of precipitates in the topography measurement and the smaller domains in the magnetic domain image. The STEM study (Fig. 2) has shown that the lines of particles are indeed the VC precipitates.

**Figure 8:**
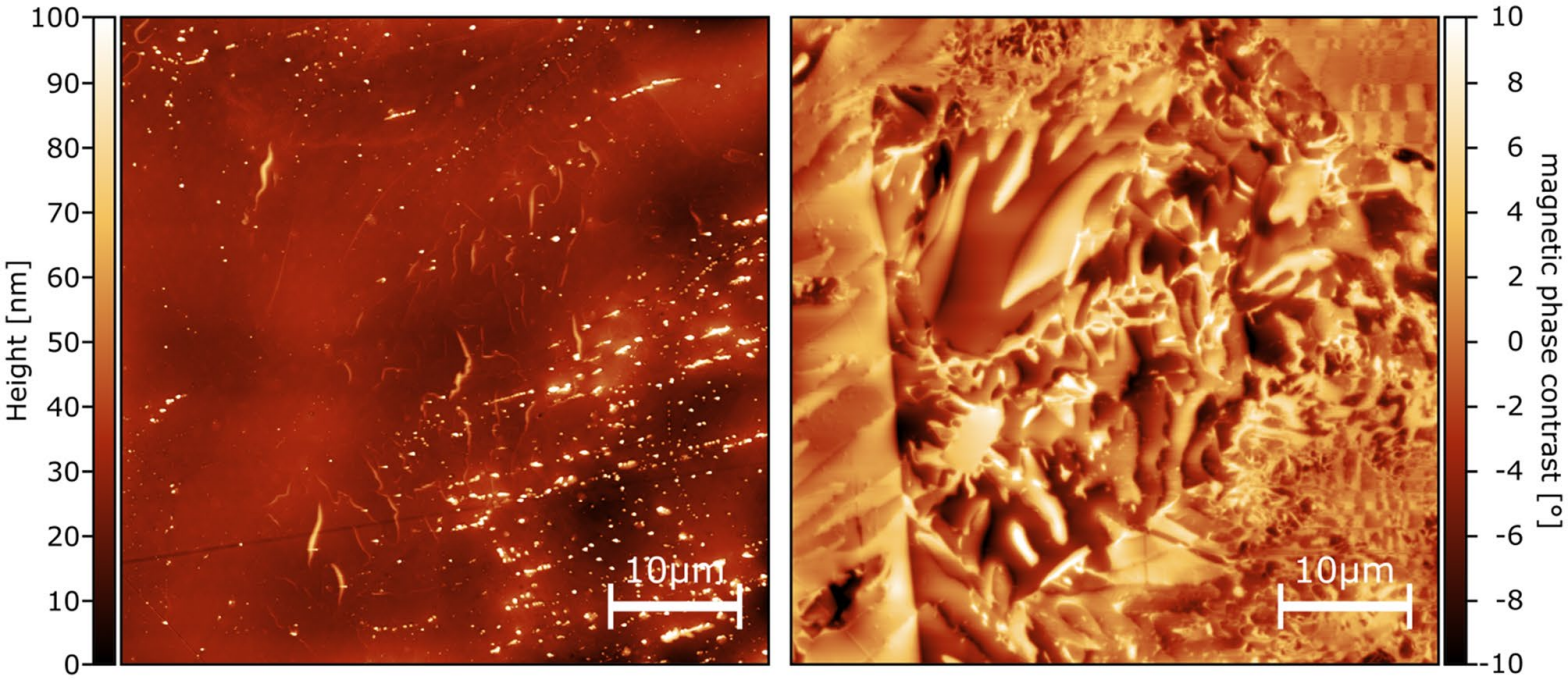
Magnetic force microscopy measurement of the Mn_55_Al_45_C_2_(VC)_1_ sample. Left image shows the topography with clear visible lines of precipitates. The right image illustrates the magnetic domains and a correlation between small domains and the location of the precipitates can be seen.

Comparing the location of the smaller and larger domains in the magnetic contrast image in Fig. 8 with the location of the precipitates in the topography, a clear correlation between smaller domain sizes and the location of precipitates can be drawn. This further corroborates the beneficial effect of the VC precipitates on the magnetic properties.

**Figure 9:**
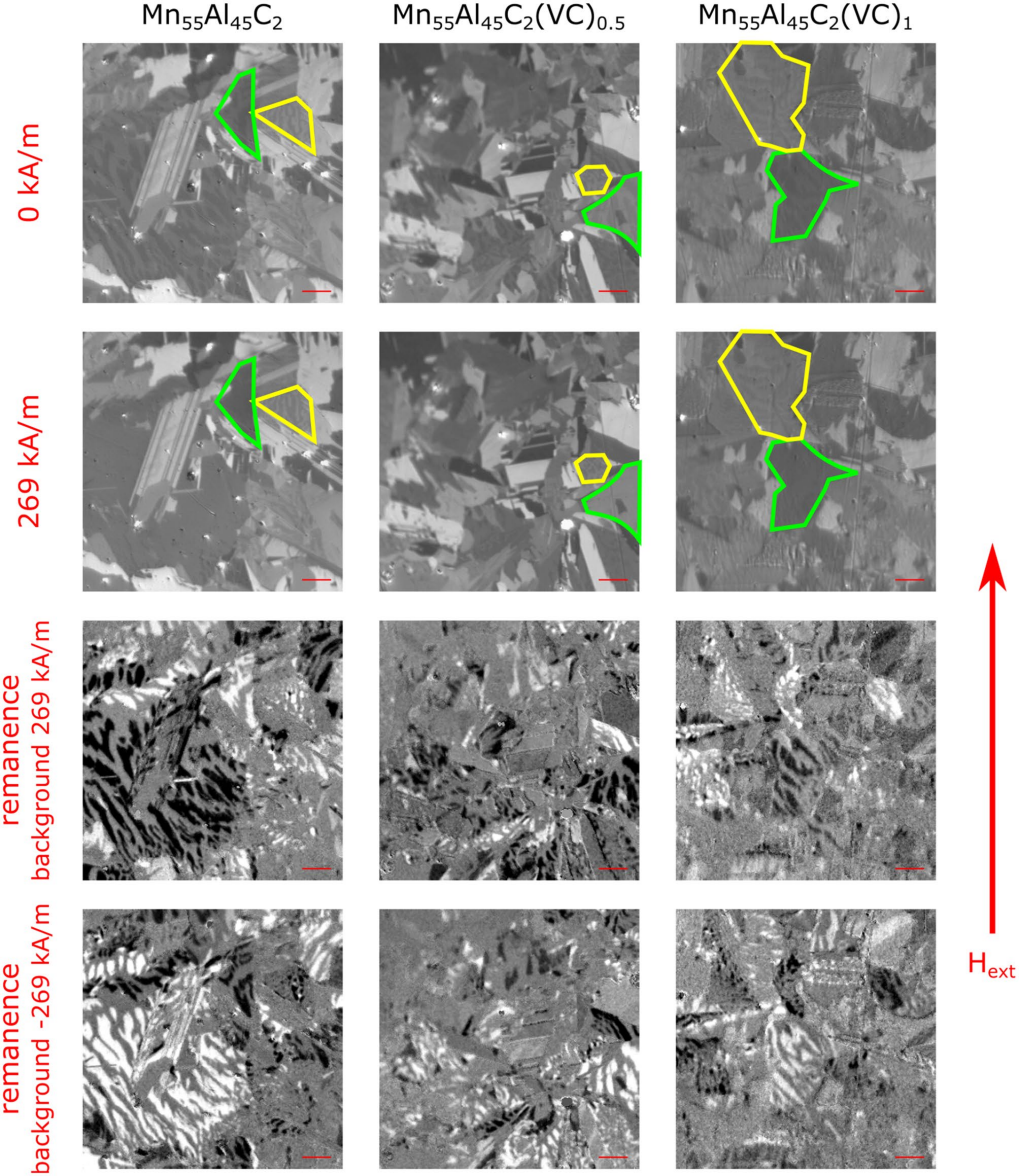
Kerr microscopy investigation of as-cast Mn_55_Al_45_C_2_, Mn_55_Al_45_C_2_(VC)_0.5_ and Mn_55_Al_45_C_2_(VC)_1_ alloys. The first row are measurements taken at 0 kA/m, while the second row shows images taken at an applied field of 269 kA/m. In all samples, grains can be identified which can be saturated (marked green) and which cannot be saturated (marked yellow). Row three and four illustrate the domain changes coming from 269 kA/m to 0 kA/m (third row) and coming from -269 kA/m to 0 kA/m (fourth row). As expected the domains in the grains are reversing their orientation (black to white and vice versa), but no obvious pinning sites can be identified. The images are cut-outs of the bigger measurements and represent 90 × 90 µm^2^, i.e. the same size as the MFM images in Fig. 8; the red scale bar illustrates 10 µm.

To investigate the domain structure under applied field we performed Kerr microscopy studies on the polished surface of the as-cast pieces of Mn_55_Al_45_C_2_, Mn_55_Al_45_C_2_(VC)_0.5_ and Mn_55_Al_45_C_2_(VC)_1_. The measurements were performed using the polar and longitudinal magneto-optical Kerr effect (MOKE); therefore, we are sensitive to magnetization components both out-of- plane and parallel to the applied in-plane magnetic field. The investigated as-cast samples have various orientations of the grains. Hence, a preferred anisotropy axis could not be identified and each grain had an almost unique response to the applied magnetic field. Figure 9 shows representative locations for each of the three samples. While the first row illustrates the MOKE images without any applied field, the second row shows the same location under the maximum applied field of 269 kA/m. The microstructure of the samples is dominating these images, but in several grains the domain structure can be identified. In each of the three samples, grains could be identified which saturated with the applied field of 269 kA/m. Examples are marked with a green border around that specific grain. Additionally, there are also grains which could not completely be saturated, which are marked by yellow borders. A plausible reason for this vastly different behavior is the random orientation of the grains in the as-cast samples. Therefore, the anisotropy axis within saturated grains is more or less aligned with the applied field direction.

Another observation based on the microstructure is that the grain size seems to be reduced for the samples Mn_55_Al_45_C_2_(VC)_0.5_ and Mn_55_Al_45_C_2_(VC)_1_ in comparison with Mn_55_Al_45_C_2_. But this observation cannot be quantified using the Kerr microscopy studies, as the domain structure visible in the images will interfere in any grain size analysis.

The lower two rows in Fig. 9 are typical domain contrast images recorded with a Kerr microscope. To receive a clear domain contrast, the Kerr image at a certain applied magnetic field is subtracted from the image at a different field. The resulting image illustrates the changes in the domain structure between the two applied fields. Therefore, we can investigate the domain dynamics by applying the magnetic field step wise. While increasing it from 0 kA/m up to 269 kA/m, we notice in all three samples that the domains change similarly. After reaching the maximum applied field at 269 kA/m we reduce the field stepwise, first down to remanence (0 kA/m) and then continuing to -269 kA/m, and ending the hysteresis loop again at 0 kA/m. Videos of these measurements can be found in the Supplementary materials. The third row in Fig. 9 shows the corresponding domain changes going from 269 kA/m to 0 kA/m, while the fourth raw illustrates the domain changes going from -269 kA/m to 0 kA/m. It is obvious that the black and white domains in row 3 are inverted in row 4. But at a closer examination of the domain change structure, it becomes clear that there is no evidence of strong pinning in the investigated samples, as there is no complete inversion of the domains. Only the expected reversing of the domain preference is visible, revealing more white than black domains.

These results from the domain dynamics are expected, as there are no big differences between the samples in the magnetization measurements for the available field range of the Kerr microscope (Fig. 5 and Table 1).

## Conclusions

The effects of the nano-VC on the microstructure and magnetic properties of Mn_55_Al_45_C_2_ have been investigated by various techniques. It was shown that the nano-VC containing Mn_55_Al_45_C_2_(VC)_*x*_ (*x* = 0.25, 0.50 and 1.0) samples were purer in τ-phase in comparison to the reference (≥ 94 wt.% vs. 85 wt.% of -phase, respectively). Metallography studies demonstrate a microstructure with nanometer sized inclusions of carbides. The addition of nano-VC significantly decreases the amount of small angle grain boundaries as compared to Mn_55_Al_45_C_2_. In relation to this, the presence of twin boundaries with angles of 75˚ and 85˚ corresponding to <100> and <110> twins, respectively, increases with the addition of nano-VC.

For nano-VC contents above 0.25 at.%, a clear increase of the coercive force is observed, from 57 to 71 kA/m. The optimum appears to be for 0.5 at.% nano-VC which shows a 25% increase in coercive force without losing any saturation magnetization. This independent increase in coercivity is believed to originate from the nano-VC reducing the overall magnetic domain size. Also, as there is a weaker than usual correlation of τ-phase purity and magnetization in these alloys, it is concluded that saturation magnetization is here not only determined by the amount of τ-phase, but also the amount of carbon, which has been reported to have effects on saturation magnetization^14,32^. Comparing carbon free τ-phase MnAl to carbon substituted τ-phase MnAl show that some amount of carbon is beneficial for saturation magnetization, while too much lowers the saturation magnetization^14^. This has also been reported to decrease K_1_ and T_C_^14^. However, all of this is further complicated by the fact that atomic order, carbon content and *c*/*a* ratio (which are all interacting) varies in these samples. Separating these effects from each other is however outside of the scope of this work.

From magnetic force microscopy, we can conclude that nano-VC precipitates reduce the domain size in Mn_55_Al_45_C_2_. This effect can be caused by nucleation of magnetic domain walls by the precipitates or being an indirect effect of a reduced grain size, but most likely it is a combined effect of both.

Kerr microscopy studies revealed that there are no measurable differences in the dynamic response to the applied magnetic field for the investigated Mn_55_Al_45_C_2_, Mn_55_Al_45_C_2_(VC)_0.5_ and Mn_55_Al_45_C_2_(VC)_1_ samples. This finding is in good agreement with the magnetization measurements. Additionally, we can see a large variety of grain orientations leading to a vastly different response of the magnetic domains to the applied field. However, we cannot identify traces of strong pinning within a single grain in neither the Mn_55_Al_45_C_2_(VC)_0.5_ or Mn_55_Al_45_C_2_(VC)_1_ sample.

Overall, we can conclude that addition of nano-VC could be an interesting route to increase the coercive force of MnAl, without sacrificing saturation magnetization. However, before this can be realized, much deeper investigations on the effects of inclusions of nanoparticles in MnAl are needed.

## Supplementary Information

Supplementary Figures.

Supplementary Video 1.

Supplementary Video 2.

Supplementary Video 3.
