# Supplementary material for: Influence of nano-VC on the structural and magnetic properties of MnAlC-alloy
Source: Sci Rep. 2021 Jul 14;11:14453. doi: 10.1038/s41598-021-93395-2 (PMC8280156; doi:10.1038/s41598-021-93395-2)
Supplement: Supplementary file 1 — Supplementary Figures. [file 41598_2021_93395_MOESM1_ESM.docx]

**Influence of nano-VC on the structural and magnetic properties of MnAlC-alloy**

V. Shtender^1,*^, H. Stopfel^2,3^, D. Hedlund^2^, D. Karlsson^1^, R. Pothala^3^, F. Olsson^4^,

G. Andersson^3^, P. Svedlindh^2^ and M. Sahlberg^1^

*^1^Department of Chemistry – Ångström Laboratory, Uppsala University, Box 538, 751 21, Uppsala, Sweden*

*^2^Department of Materials Science and Engineering, Uppsala University, Box 35, 751 03 Uppsala, Sweden*

*^3^Department of Physics and Astronomy, Uppsala University, Box 516, 751 20 Uppsala, Sweden*

*^4^Höganäs AB, Bruksgatan 35, 263 83, Höganäs, Sweden*

*Corresponding author:

[vitalii.shtender@kemi.uu.se](mailto:vitalii.shtender@kemi.uu.se)

Supplementary information


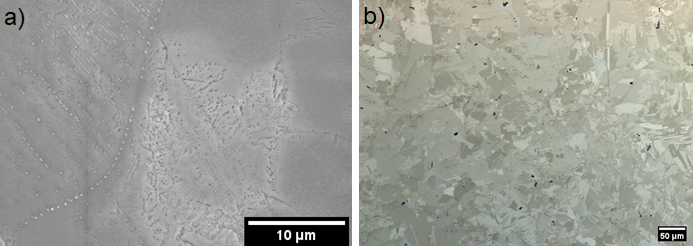


Figure S1. SEM (a) and LOM (b) images of the etched and nonetched Mn_55_Al_45_C_2_(VC)_1_ alloy, respectively.


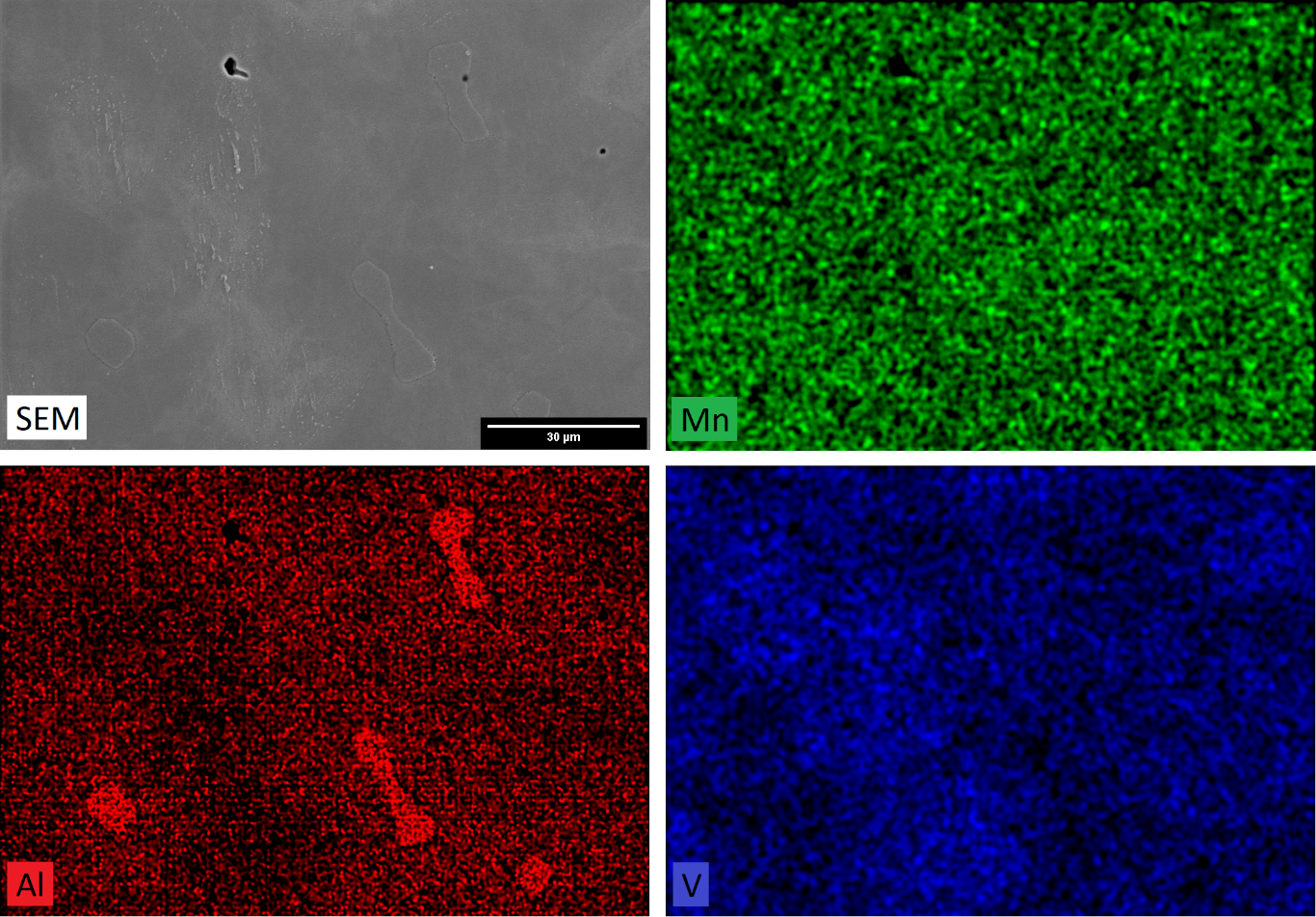


Figure S2. EDS maps of Mn_55_Al_45_C_2_(VC)_1_ showing a homogeneous alloy with Al-enriched areas which are related to the γ_2_-phase. Some chemical segregations of V in areas where the concentration of Mn seems higher and Al less can be noticed.


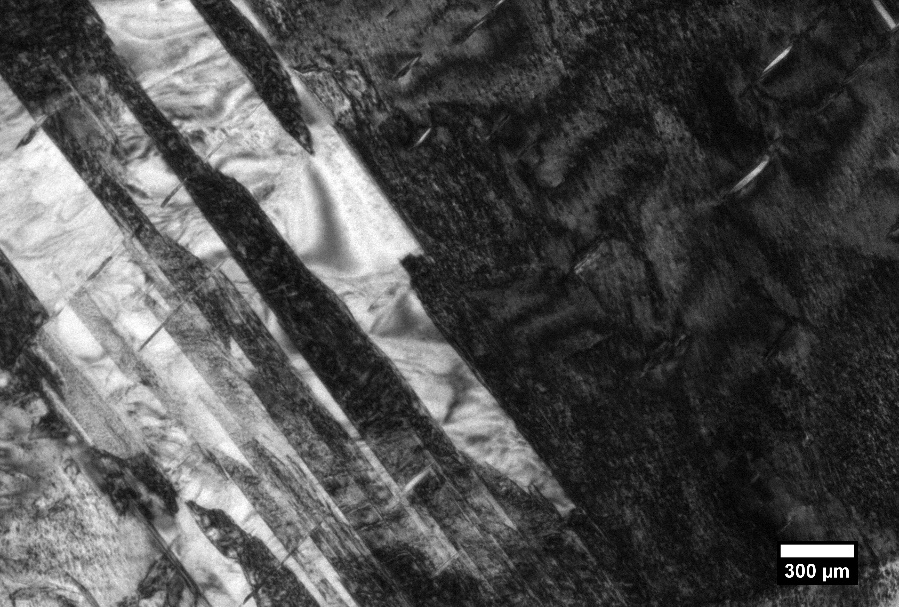


Figure S3. TEM image of the Mn_55_Al_45_C_2_(VC)_1_ sample, showing preferred orientation of VC-particles in regard to the τ-phase and across twin boundaries
